# Strong correlation of novel sleep electroencephalography coherence markers with diagnosis and severity of posttraumatic stress disorder

**DOI:** 10.1038/s41598-018-38102-4

**Published:** 2019-03-12

**Authors:** Mo H. Modarres, Ryan A. Opel, Kristianna B. Weymann, Miranda M. Lim

**Affiliations:** 1 Brain Rehabilitation Research Center, Malcom Randall VA Medical Center, Gainesville, Florida USA; 2 Sleep Disorders Clinic, VA Portland Health Care System, Portland, Oregon USA; 3 School of Nursing, Oregon Health & Science University, Portland, Oregon USA; 4 Department of Neurology, Oregon Health & Science University, Portland, Oregon USA; 5 Department of Behavioral Neuroscience, Oregon Health & Science University, Portland, Oregon USA; 6 Department of Medicine, Oregon Health & Science University, Portland, Oregon USA; 7 Oregon Institute of Occupational Health Sciences, Oregon Health & Science University, Portland, Oregon USA

## Abstract

Objective biomarkers of the presence and severity of posttraumatic stress disorder (PTSD) are elusive, yet badly needed. Electroencephalographic (EEG) coherence represents a promising approach to identifying and understanding brain biomarker activity in PTSD. Overnight polysomnography data containing EEG across sleep and wake states was collected in n = 76 Veterans with and without PTSD from a single site under IRB approval. Brain coherence markers (BCM) were calculated from EEG signals using a novel approach to produce one index for PTSD diagnosis (*PTSD*_*dx*_), and another index for PTSD severity (*PTSD*_*sev*_). *PTSD*_*dx*_ showed strong sensitivity to the presence of PTSD in the awake state, during non-rapid eye movement (NREM) stage N2 sleep, and in a hybrid BCM incorporating both awake and NREM sleep states. *PTSD*_*sev*_ showed a strong correlation with PTSD symptom severity (using the PTSD Checklist 5, or PCL5 survey) in the awake state, during N2 sleep, and in a hybrid BCM incorporating both awake and NREM sleep states. Thus, sleep EEG-based brain coherence markers can be utilized as an objective means for determining the presence and severity of PTSD. This portable, inexpensive, and non-invasive tool holds promise for better understanding the physiological mechanisms underlying PTSD and for tracking objective responses to treatment.

## Introduction

Post-traumatic stress disorder (PTSD) is a chronic and disabling neuropsychiatric disorder that is characterized by severe sleep disturbances, avoidance behaviors, physiological hyper-arousal, and re-experiencing symptoms following exposure to a traumatic event^1^. Epidemiologic studies have shown that nearly 56% of people will experience a psychologically traumatic event and between 7–12% of individuals will meet criteria for PTSD during their lifetimes^2–5^. In the United States military, Veterans have an even higher risk of developing PTSD compared to the civilian population, with the lifetime prevalence of PTSD estimated at 19% for Vietnam-era Veterans^6^. Similar patterns are observed among OIF/OEF Veterans: Approximately 17% of active duty soldiers and 25% of OIF met criteria for PTSD 3–6 months post-deployment^7^.

The current practice for diagnosing of PTSD primarily relies on subjective clinical assessments by the clinicians and patients’ self-reports. Subjective evaluation of PTSD symptom severity is also an integral part of the current treatment approaches for PTSD. An objective and neuro-physiologically-based method for directly assessing brain function in PTSD is currently unavailable, but badly needed in order to improve diagnostic specificity, as well as to track response to treatment, of PTSD. This need was highlighted by recent recommendations from the Institute of Medicine, National Academy of Science (IOM-NAS), which conducted a comprehensive assessment of the current PTSD diagnosis and treatment methods and identified potential shortcomings of the current diagnostic and treatment techniques^8^. A major recommendation by the IOM-NAS was to fill the urgent need for development of methods for the more precise and objective diagnosis of PTSD and its severity level, for the objective and faster evaluation of treatment efficacy, and for the ability to predict who might be at risk of relapse. The IOM-NAS report emphasizes that in order for a biological and/or physiological marker to be considered of value in the diagnosis of PTSD, the marker must first require excellent sensitivity and specificity in distinguishing persons who have and do not have PTSD, and second, be correlated with severity of PTSD.

A number of reported brain imaging studies have shown altered brain activity and connectivity in PTSD in the ventromedial prefrontal cortex (vmPFC), insula, amygdala, and hippocampus^9–12^. Functional connectivity in PTSD is also altered among several brain regions, such as the parietal, temporal, and central regions of the cortex^13–19^. Collectively, these studies postulate that abnormal neural connections and coherence between regions may underlie the pathophysiology of PTSD, and that a stereotypical pattern of connectivity or coherence may contribute to the severity of symptoms experienced.

Alterations in electroencephalography (EEG) and magneto-encephalography (MEG) signals have been previous associated with PTSD, compared with control subjects^20–24^. These studies typically examined subjects in the awake state, some using task-based approaches as well as in a state of quiet wakefulness (e.g., resting state functional connectivity). Results from these studies indicated alterations in both local EEG and MEG activity, as well as inter- and intra-hemisphere connectivity and synchronicity. One specific finding of note is from Lee *et al*.^17^, an EEG study that showed decreased resting-state functional connectivity in PTSD compared with control subjects. Furthermore, they also found that functional connectivity measures were significantly correlated with PTSD symptom severity.

PTSD is also associated with an extraordinarily high prevalence of sleep disturbances, to the point that some have proposed that sleep disturbances are a hallmark feature of the disorder^20–31^. Over 70% of civilians and Veterans with PTSD have reported persistent and severe nightmares and disturbed sleep^21^. Further evidence of the strong association between PTSD and sleep disturbance was provided by Germaine *et al*., who studied 367 people with PTSD and found that the severity of PTSD was closely correlated with the severity of sleep disturbances^22^. More importantly, evidence suggests that sleep disturbance appears before the onset of PTSD; therefore, disturbed sleep could be an early marker in development of PTSD contributing to maladaptive stress^29–31^. One such comprehensive review by Spoormaker and Montgomery^27^, which reviewed a number of clinical studies that provided evidence for the occurrence of sleep disturbance prior to the onset of PTSD symptoms, highlighted several findings from Mellman *et al*.^24^. Of four relevant Mellman *et al*. studies, the first found that REM sleep disturbances found within one month of traumatic injuries in 21 subjects were predictive of the severity of their PTSD symptoms 6 weeks later. A second study^32^ reported that initial sleep disturbances in n = 71 subjects within one month of motor vehicle accidents (MVA) were predictive of the PTSD development that occurred 6 months later. In a third, larger study of n = 102 MVA survivors, self-reported sleep disturbances were predictive of PTSD one year later^23^. Finally, a fourth study by Mellman *et al*.^33^ reported that the nightmares occurring within one month after civilian trauma was predictive of the severity of PTSD symptoms 6 weeks later.

Based on this previous literature, we hypothesized that a brain-based biomarker sensitive and specific to PTSD could be derived from dynamic measures of synchronous activity among the regions of cerebral cortex during specific stages of sleep. Thus, we analyzed electroencephalography (EEG) from overnight polysomnography, containing extended periods of sleep and wakefulness, from n = 38 Veterans with PTSD compared to n = 38 age-matched Veterans without PTSD.

## Methods and Materials

### Human Subjects

**Figure 1:**
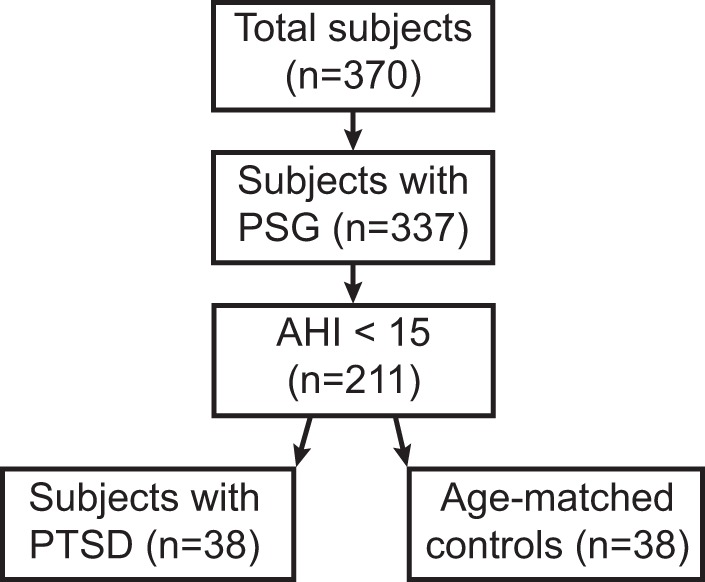
Consort diagram showing inclusion/exclusion criteria. PSG = Polysomnography; AHI = apnea-hypopnea index; PTSD = posttraumatic stress disorder.

All participants provided informed consent under VA Portland Health Care System (VAPORHCS) Institutional Review Board approval (MIRB #3641), and the study was conducted in accordance with the ethical guidelines of the Belmont Report. Participants were consented upon referral to the VAPORHCS Sleep Clinic between May 2015 and November 2016 (n = 370). Subjects with in-lab, overnight polysomnography (PSG; *n* = 337) were included in the initial study population. Participants were excluded if they had an apnea-hypopnea index of fifteen or greater (*n* = 126; Fig. 1). Remaining subjects were assessed for the presence of PTSD via the PTSD Checklist for DSM-V survey (PCL-5, see definition below). Participants who met criteria for PTSD (*n* = 38) were then age-matched to *n* = 38 non-PTSD controls (Table 1).

**Table 1:** Participant Demographics. Data are presented as mean ± SEM for continuous variables and count (%) for categorical variables.

	Control (n = 38)	PTSD (n = 38)	Statistic	*P* Value
Age, years	55.6 ± 2.7	50.4 ± 2.6	1.41	0.163
BMI	31.1 ± 1.1	31.0 ± 1.3	0.04	0.965
Sex (% male)	35 (92%)	28 (87%)	0.14	0.709
AHI	6.7 ± 0.7	6.8 ± 0.8	0.10	0.921
PCL-5, score	11.2 ± 1.3	49.9 ± 1.9	16.81	<0.001

AHI = apnea-hypopnea index, PCL-5 = Post-Traumatic Stress Disorder Checklist for DSM-V.

### Source of Data

#### Polysomnography

**Figure 2:**
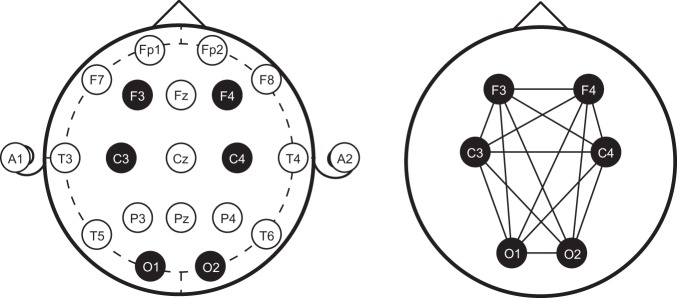
Electrode locations of International 10–20 system for EEG (electroencephalography) recording. PSG data were recorded at standard clinical sleep EEG locations according the International 10–20 EEG placement sites. The EEG Montage for the sleep study consisted of the following, highlighted leads: Frontal: F3 and F4, Central: C3 and C4, and Occipital: O1 and O2. The panel on the right indicates all possible EEG coherence pairs for computation.

Subjects in the data repository underwent in-lab overnight polysomnography (PSG) using Polysmith (NihonKohden, Japan). Six scalp electrodes were placed at F3, F4, C3, C4, O1, and O2 per the 10–20 system of EEG placement (Fig. 2). Following the conclusion of the study, an American Academy of Sleep Medicine (AASM)-accredited polysomnographic technician manually performed standard sleep staging analysis for each 30-second epoch duration according to the standard clinical criteria. Each 30-second epoch of data was scored as one of the five sleep stages (Awake [W], Rapid Eye Movement [REM], non-REM [NREM] stages N1, N2, and N3). Staging of each PSG was additionally validated by a board-certified sleep physician blinded to PTSD status. In the event that there was nonconvergence between scorers, the board-certified sleep physician made the final call.

#### Post-Traumatic Stress Disorder Checklist

PTSD symptom severity using the PTSD Checklist (PCL-5) was obtained at the time of the overnight PSG. The PCL-5^34^ is a 20-item self-report (score range from 0–80) which assesses the twenty PTSD symptoms outlined in the Diagnostic and Statistical Manual of Mental Disorders, 5th Edition (DSM-V)^35^. Diagnosis of PTSD was determined by 1) meeting the PCL-5 Cluster Criteria (as follows: The questions are subdivided into four subscales, or clusters: Cluster B (Intrusion; 1-5), Cluster C (Avoidance; 6-7), Cluster D (Mood and Cognition; 8-14), and Cluster E (Arousal Activity; 15-20). Cluster A was not administered in this study. The PCL-5 cluster criteria require subjects to rate 1 B item, 1 C item, 2 D items, and 2 E items as “2-Moderately” or higher), and 2) scoring a total of 33 or greater^34^. Cronbach alpha in our sample was 0.98 [0.97–0.98], consistent with previously reported values^36^.

### Computation of First Pass Brain Coherence Markers (*BCM*)

EEG signal processing from overnight PSG was performed on each of the 6 EEG leads from each subject. PSG records were deidentified and exported as EDF files. Spectral power coherence between pair combinations of EEG was calculated to produce candidate BCM using MATLAB (MathWorks, Natick, MA).

Coherence is a normalized quantity (index spans from zero to one) that reflects the degree of association or coupling of the spectral power levels in a pair of EEG waveforms (from two scalp sites) and for a given frequency band. Formally, coherence is a generalization of correlation analysis and is computed as the magnitude of normalized cross-power spectrum^37^ of a pair of simultaneously recorded EEGs from two separate scalp locations^38^.

Denoting EEG signals recorded at two scalp locations as *x* and *y*, coherence between *x* and *y* is defined per (1), where ${G}_{xy}(f)$ is the cross-spectral density between *x* and *y* at the frequency *f*, and where ${G}_{xx}(f)$ and ${G}_{yy}(f)$ are the auto-spectral density (at frequency *f*) of *x* and *y*, respectively.

1 $${C}_{xy}(f)=\frac{{|{G}_{xy}(f)|}^{2}}{{G}_{xx}(f){G}_{yy}(f)}$$

Such EEG coherences reflect the degree of coupling and functional association between two brain regions^38^ and can be computed for any specific frequency bands of EEG (e.g., six canonical EEG frequency bands: Delta (δ): 1–3.5 Hz; Theta (θ): 4–7.5 Hz; Alpha (α): 8–12 Hz; Sigma (σ): 13–16 Hz; Beta (β): 16.5–25 Hz; and Gamma (γ): >30 Hz). Coherence can also be computed over any frequency bands, including discrete frequency levels (single Hz). Additionally, an extra dimension of sleep vs. wake states was also included in the analysis.

Coherence functions (1) were computed from pairs of the six EEG electrodes, which produced a total of 15 coherence data records for all of the electrode pairs: 9 inter-hemispheric pairs (O1-O2, C3-C4, F3-F4, O1-C4, O2-C3, C3-F4, C4-F3, O1-F4, and O2-F3), and 6 intra-hemispheric pairs (O1-C3, O2-C4, C3-F3, C4-F4, O1-F3, and O2-F4). The coherence values were computed for the entire sleep study using 5-second sliding windows that were overlapped by 1 second. The frequency band spanned from 0.2 Hz to 50 Hz with 0.2 Hz resolution (1/5 seconds), thus, the coherence values were computed at the following 250 frequencies: 0.2, 0.4, 0.6, 0.8, 1.0, 1.2, … 50 Hz.

Vectors of *BCM* (2), where ${C}_{{e}_{i},{e}_{j}}(t,f)$ is the coherence between the EEG signals *e*_*i*_ and *e*_*j*_. from two different scalp electrode locations,

2 $${\boldsymbol{BCM}}({e}_{i},{e}_{j},{F}_{range},{T}_{period})=\frac{1}{length({T}_{period})\cdot length({F}_{range})}{\sum }_{t={T}_{period}}{\sum }_{f={F}_{range}}{C}_{{e}_{i},{e}_{j}}(t,f)$$

were constructed using all 15 electrode pairs, an inclusive 1 Hz frequency bandwidth around the entire frequency range, and over each of the five stages of sleep study (W, REM, N1, N2, N3). For example, *BCM* (O1, O2, *F*_*range*_ = [9.4, 9.6, 9.8, 10.0, 10.2, 10.4], *T*_*period*_ = N2) corresponds to the coherence between the left and right occipital EEG, integrated over 9.4 and 10.4 Hz frequency range, and averaged for the N2 sleep period. Thus, *BCM* is computed as the mean value of coherence function (1) over frequency range ${F}_{range}$ and averaged over time-period $\,{T}_{period}$.

The ratios of *BCM* (3), referred to as *BCMr*, where *BCMi* is defined in (2), was defined as:

3 $${\boldsymbol{BCM}}r=\frac{{\boldsymbol{BC}}{{\boldsymbol{M}}}_{1}}{{\boldsymbol{BC}}{{\boldsymbol{M}}}_{2}}=\frac{{\boldsymbol{BCM}}({e}_{i,1},{e}_{j,1},{F}_{range,1},{T}_{period,1})}{{\boldsymbol{BCM}}({e}_{i,2},{e}_{j,2},{F}_{range,2},{T}_{period,2})}$$

*BCMr* were computed for all 15 electrode pairs, all possible inclusive 1 Hz frequency ranges (from 0.2 to 50 Hz), and all five sleep stages. Thus, for each subject, a total of n=128,625 *BCMr* were calculated. This number is the product of 105 electrode pair ratios (using 15 EEG pairs) $\times$ 245 total frequency ranges $\times$ five sleep stages.

The vector of all possible BCMr for PTSD diagnosis was reduced to a vector of candidate of 105 BCMr as follows: For a given sleep state, and a particular BCMr that corresponded to two sets of EEG pairs (4 EEG electrodes, one pair of EEG coherences for the numerator coherence and one pair for the denominator EEG coherence), the vector of BCMr for all possible 1 Hz frequency bands were computed. The best BCMr was thus chosen based on maximizing the mean difference between the PTSD and control group (i.e., maximizing the F ratio). A total of 105 candidate BCMr were calculated corresponding to a total 105 possible ratios constructed from 15 EEG pairs.

Similarly, the vector of all possible BCMr for determining PTSD severity was reduced to a vector of 105 candidate BCMr as follows: For a given sleep state, and a particular BCMr that corresponded to two sets of EEG pairs, the vector of BCMr for all possible 1 Hz frequency bands were computed. The best BCMr was thus chosen based on maximizing the R^2^ regression coefficient with PCL-5 as the dependent variable. Theses candidate *BCMr* for calculating indices for diagnosis and severity of PTSD (detailed below) were not corrected for multiple comparisons, as these were utilized as a first pass to generate promising candidates for subsequent linear combination, as described below.

### Computation of *PTSD*_*dx*_ and *PTSD*_*sev*_

The best vectors of *BCMr* were then linearly combined to produce two separate indices, associated with the *diagnosis* (*PTSD*_*dx*_) and *severity* (*PTSD*_*sev*_) of PTSD, respectively. Indices were calculated for all of the 76 subjects for *PTSD*_*dx*_, and the 38 subjects with PTSD for *PTSD*_*sev*_. For each sleep stage, *BCMr* were pooled from every subject in the relevant patient sample. For *PTSD*_*dx*_, stepwise logistic regressions were performed, where *BCMr* served as the independent variable and PTSD status was used as the dependent variable. For *PTSD*_*sev*_, correlation analyses were performed between *BCMr* and PCL-5. Using these set of candidate *BCMr*, linear discriminant analysis was performed where the set of candidate *BCMr* were linearly combined to produce either the best group discrimination (*PTSD*_*dx*_; evaluated by minimizing R^2^ and *F* statistics) or strongest correlation level with PCL-5 (*PTSD*_*sev*_).

Final *BCM* of *PTSD*_*dx*_ were evaluated using univariate means analysis corrected for multiple (e.g., n = 128,625) comparisons using Bonferroni correction. Final *BCM* of *PTSD*_*sev*_ was evaluated using correlation corrected for multiple (e.g., n=128,625) comparisons using Bonferroni correction.

To evaluate precision of *PTSD*_*dx*_ and *PTSD*_*sev*_, accuracy (4) was defined as the following:

4 $${\rm{Accuracy}}=100\times (1-\frac{\,\mathrm{No}.\,{\rm{of}}\,{\rm{False}}\,{\rm{Positives}}+{\rm{No}}.\,{\rm{of}}\,{\rm{False}}\,\mathrm{Negatives}\,}{{\rm{Total}}\,{\rm{No}}.\,{\rm{of}}\,{\rm{Subjects}}}),$$

where False Positives are defined as the number of Control subjects (without PTSD) that could be mislabeled as PTSD, and False Negatives are the number of PTSD subjects that could be mislabeled as Control (without PTSD).

### Computation of *Hybrid*_*PTSD*_*dx*_ and Hybrid_*PTSD*_*sev*_

Because our analyses showed that *PTSD*_*dx*_ and *PTSD*_*sev*_ obtained from each individual awake and sleep state were highly significant in identifying and tracking severity of PTSD, we hypothesized that simultaneous incorporation of the *PTSD*_*dx*_ and *PTSD*_*sev*_ from all three vigilance states would have a multiplicative effect, and produce accuracies even higher than those markers obtained from individual sleep states.

We thus defined a hybrid *PTSD*_*dx*_ marker as follows:

5 $${Hybrid}\_{PTS}{{D}}_{{dx}}=PTS{D}_{dx}(W)\times PTS{D}_{dx}{(N1)}^{k1}\times PTS{D}_{dx}{(N2)}^{k2}$$

where W, N1, and N2 refer to awake, stage N1 sleep, and stage N2 sleep, respectively. An optimization procedure with an objective function associated with maximizing the degree of separation between PTSD and Control groups produced power coefficients of k1 = 0.1 and k2 = 0.5.

## Results

### *BCM* associated with the diagnosis of PTSD

#### Awake State

First pass, uncorrected statistical t-test comparisons of the eight best individual *BCMr*, named *BCMr*_1_–*BCMr*_8_, were performed between the PTSD and Control groups (Supplementary Table S1). Markers *BCMr*_1,_ *BCMr*_3,_ *BCMr*_5,_ and *BCMr*_6_ were larger in individuals with PTSD compared to Controls, while the other four markers were reduced in the PTSD group. The right column of Supplementary Table S1 shows a diagram of the coherence pairs that comprised the *BCMr*, where the thickness of the line connecting the electrode pairs is proportional to the number of occurrences of that particular *BCM*. For example, the thick line *BCM* between O2 (right occipital) and C4 (right central) is indicative of 5 occurrences of this marker (as part of *BCMr*_3,_ *BCMr*_5,_ *BCMr*_6,_ and *BCMr*_7_). This diagram shows that the coherence between the right hemisphere occipital lobe (O2) and the right central site C4 strongly contributed to the markers that were able to separate control from PTSD group. Other strong connections appear to be between the left and right frontal lobes (F3 and F4), as well as another interhemispheric connection between the left central and right frontal lobe.

**Figure 3:**
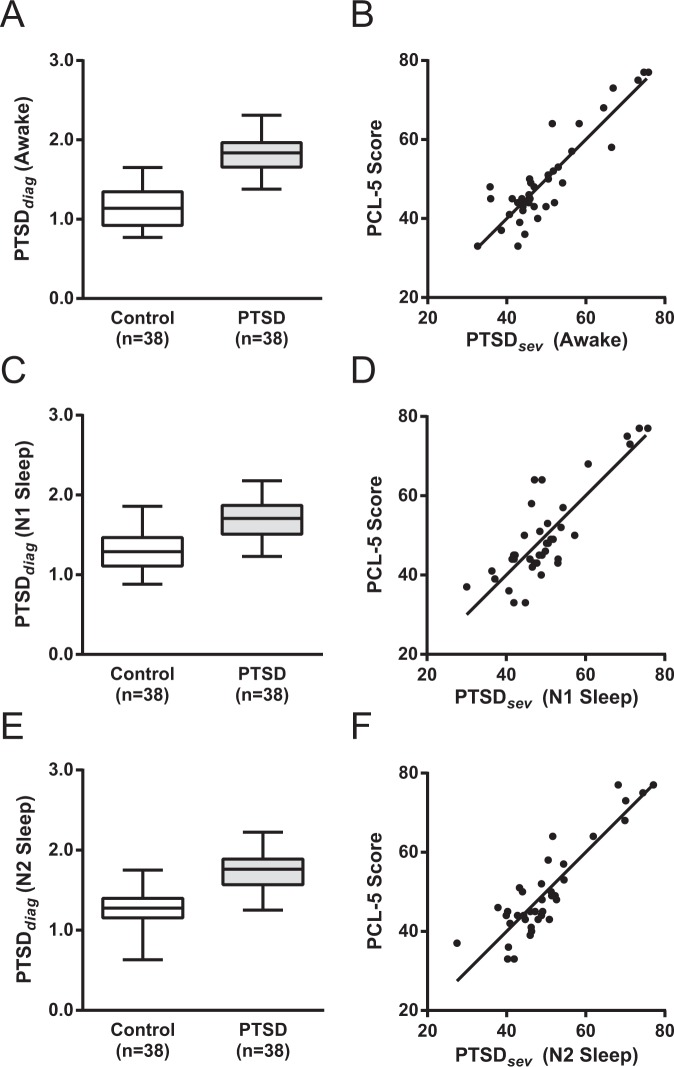
Comparison of *PTSD*_*dx*_ between PTSD and Control groups (Left Column); PCL-5 vs *PTSD*_*sev*_ of PTSD group (Right Column). Left column: Boxplots of *PTSD*_*dx*_ brain coherence marker for control and PTSD groups in wakefulness (Panel A), N1 Sleep (Panel C), N2 Sleep (Panel E). On each box, the central mark is the median, the edges of the box are the 25th and 75th percentiles, the whiskers extend to the most extreme data-points the algorithm considers to be not outliers, and the outliers are plotted individually. Right Column: Scatter plots of *PTSD*_*sev*_ vs PCL-5 in groups in wakefulness (Panel B), N1 Sleep (Panel D), N2 Sleep (Panel F), as well as the regression line for each state.

*BCMr*_1_ – *BCMr*_8_ were linearly combined to produce a single, final, variable known as *PTSD*_*dx*_ that significantly distinguished Control from PTSD in the awake state (Control = 1.17 ± 0.26, PTSD = 1.83 ± 0.22; t_74_ = 12.1, *P* < 0.0001, unpaired t-test) (Fig. 3A). A regression was performed using *PTSD*_*dx*_ as the independent variable against a binary variable belonging to PTSD or control groups serving as the dependent variable. The results of the regression analysis show an R^2^ (coefficient of determination) of 0.67 that was highly significant (*F*_1,36_ = 145, *P*_*adj*_ < 10^−5^; Pearson’s correlation).

To evaluate the precision of *PTSD*_*dx*_ for the separation of the two groups, accuracy was calculated as 76% during the awake state.

#### N1 Sleep

First pass, uncorrected statistical t-test comparisons of the two best individual *BCMr*, named *BCMr*_1_ and *BCMr*_2_, were performed between the PTSD and Control groups (Supplementary Table S2). Supplemental Table S2 shows the statistical t-test comparison of the two best individual *BCMr* in PTSD and Control group, which were larger in the PTSD group compared to controls. The right column shows a diagram of all the coherence pairs that were part of the *BCMr* ratios separating control from PTSD group. This diagram shows that the coherence between the right hemisphere occipital lobe (O2) and the left central site C3 strongly contributed to the markers that were able to separate controls from the PTSD group. Inter-hemispheric coherence levels between central and frontal sites also contributed to the overall diagnostic *PTSD*_*dx*_ marker.

*BCMr*_1_ and *BCMr*_2_ were linearly combined to produce the variable *PTSD*_*dx*_ during N1 sleep, which is significantly larger in PTSD compared with the control group even after correction for multiple comparisons (Control = 1.31 ± 0.26, PTSD = 1.69 ± 0.24; t_74_ = 6.8, *P* < 0.0001, unpaired t-test) (Fig. 3C). The result of the regression analysis (*PTSD*_*dx*_ independent variable, group belongings as the dependent variable) shows R^2^ = 0.39 was significant (*F* = 46, *P*_*adj*_ < 10^−3^; Pearson’s correlation).

To evaluate the precision of *PTSD*_*dx*_ for the separation of the two groups, accuracy was calculated as 34% during stage N1 sleep.

#### N2 Sleep

First pass, uncorrected statistical t-test comparisons of the five best individual *BCMr*, named *BCMr*_1_ – *BCMr*_5_, were performed between the PTSD and Control groups (Supplementary Table S2). The right column shows a diagram of all the coherence pairs that were part of the *BCMr* ratios separating control from PTSD group. This diagram shows that the coherence among the left hemisphere occipital lobe (O1), the left central site (C3), and the left frontal area (F3) strongly contributed to the markers that were able to separate control from PTSD group. Inter-hemispheric coherence levels between central and frontal sites also contributed to the overall diagnostic *PTSD*_*dx*_ marker.

*BCMr*_1_ – *BCMr*_5_ were linearly combined to produce the variable *PTSD*_*dx*_ during stage N2 sleep. *PTSD*_*dx*_ was significantly larger in PTSD compared with the control group, even after correcting for multiple comparisons (Control = 1.25 ± 0.26, PTSD = 1.75 ± 0.25; t_74_ = 8.6, *P* < 0.0001, unpaired t-test) (Fig. 3E). The result of the regression analysis (*PTSD*_*dx*_ independent variable, group belongings as the dependent variable) shows R^2^ = 0.50 that was significant (*F* = 74, *P*_*adj*_ < 10^−7^; Pearson’s correlation).

To evaluate the precision of *PTSD*_*dx*_ for the separation of the two groups, accuracy was calculated as 47% during stage N2 sleep.

### *BCM* associated with the severity of PTSD

#### Awake State

First pass, uncorrected regression correlations of the five best *BCMr* markers for PTSD symptom severity in the awake state are illustrated in Supplementary Table S3. The diagrams show that the coherence between the left hemisphere occipital lobe (O1) and the bi-hemispheric central and frontal sites (C3, F3, C4, F4) strongly contributed to the markers that were highly correlated with PCL-5. Also, interhemispheric coherences between the central and frontal lobes are another source of contribution to *PTSD*_*sev*_ marker.

*BCMr*_1_ to *BMCr*_5_ were linearly combined to produce the variable *PTSD*_*sev*_ for each individual subject. *PTSD*_*sev*_ significantly correlated with PCL-5 scores (R^2^ = 0.81, *F* = 148, *P*_*adj*_ < 10^−2^, Pearson’s correlation) (Fig. 3B).

#### N1 Sleep

First pass, uncorrected regression correlations of the two best *BCMr* markers for PTSD symptom severity during N1 sleep are illustrated in Supplementary Table S4. The diagram shows that the interhemispheric coherences between the central and frontal lobes significantly contribute to this Stage N1 *PTSD*_*sev*_ marker.

These two *BCMr* markers were linearly combined to produce the variable *PTSD*_*sev*_ for each individual subject during N1 sleep. *PTSD*_*sev*_ significantly correlated with PCL-5 scores (R^2^ = 0.7, *F* = 83, *P*_*adj*_ < 10^−5^, Pearson’s correlation) (Fig. 3D).

#### N2 Sleep

First pass, uncorrected regression correlations of the two best *BCMr* markers for PTSD symptom severity during N2 sleep are illustrated in Supplementary Table S4. The diagram in this table shows that the right intra-hemispheric coherences between the occipital and frontal lobe significantly contribute to this Stage N2 *PTSD*_*sev*_ marker. Inter-hemispheric coherences between the left and right hemisphere occipital, central, and frontal areas during stage N2 sleep also contributed to the *PTSD*_*sev*_.

These two *BCMr* markers were linearly combined to produce the variable *PTSD*_*sev*_ for each individual subject during N2 sleep. *PTSD*_*sev*_ significantly correlated with PCL-5 scores (R^2^ = 0.8, *F* = 140, *P*_*adj*_ < 10^−5^, Pearson’s correlation) (Fig. 3F).

### Hybrid *BCM* Based on the Combination of the *PTSD*_*dx*_ and *PTSD*_*sev*_ from Awake, N1 and N2 Sleep

Because our analyses above showed that *PTSD*_*dx*_ and *PTSD*_*sev*_ obtained from each individual awake and sleep state were highly significant in identifying and tracking severity of PTSD, we hypothesized that simultaneous incorporation of the *PTSD*_*dx*_ and *PTSD*_*sev*_ from all three states into a hybrid marker would have a multiplicative effect, and produce accuracies even higher than those markers obtained from individual sleep states.

**Figure 4:**
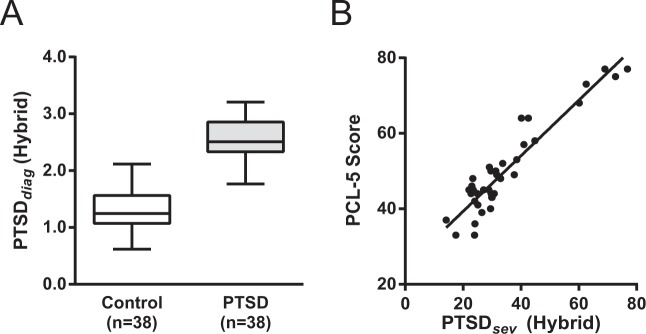
Comparison of Hybrid *PTSD*_*dx*_ and *PTSD*_*sev*_ Brain Coherence Markers. Boxplot of *PTSD*_*dx*_ for Control and PTSD subjects in the combined W, N1, and N2 (Panel A). Scatter plot of *PTSD*_*sev*_ vs. PCL-5 in the combined W, N1, and N2 (Panel B).

*Hybrid_PTSD*_*dx*_ was significantly larger in the PTSD group compared to the control group (Control = 1.34 ± 0.06, PTSD = 2.55 ± 0.07; t_74_ = 13.5, *P* < 0.0001, unpaired t-test) (Fig. 4A). Comparisons between the ANOVA and accuracy values in *Hybrid_PTSD*_*dx*_ and *PTSD*_*dx*_ are as follows: Wake, *F* = 147, 76% accuracy; N1, *F* = 46, 34% accuracy; N2, *F* = 73, 47% accuracy; Hybrid, *F* = 184, 83% accuracy, and highlight the superior performance of the hybrid marker.

*Hybrid_PTSD*_*sev*_ correlated with PCL-5 scores were highly significant (R^2^ = 0.88, *F* = 257, *P* < 0.0001) (Fig. 4B). Comparisons between the regression statistics and accuracy values in *Hybrid_PTSD*_*sev*_ and *PTSD*_*sev*_ are as follows: Wake, *F* = 148, R^2^ = 0.81; N1, *F* = 83, R^2^ = 0.70; N2, *F* = 140, R^2^ = 0.80; Hybrid, *F* = 257, R^2^ = 0.88.

These results indicate that the Hybrid markers, based on combining markers computed in awake, N1, and N2 sleep states, produce a more accurate means of assessing the presence of PTSD, and also are a more sensitive marker of the severity of PTSD symptoms.

## Discussion

We developed and evaluated a series of PTSD-sensitive neuromarkers based on changes in coherent activity measured by EEG during sleep. An innovative and unique aspect of our approach is that rather than focusing on the amplitude of the coherence function, we have based our analysis on the relative strength of the EEG coherence between one set of electrode pairs compared to the coherence level of another pair and at different EEG frequencies. Given the importance of sleep in PTSD, we have also added an extra dimension of sleep state associated with the EEG coherences at various frequency bands. Candidate neuromarkers (e.g., EEG coherence ratios) were then combined using linear regression, which produced two highly sensitive and specific indices tuned for detecting the presence and severity of PTSD across sleep-wake states.

Our study found several interesting relationships between coherence of frontal, central and occipital intra- and inter-hemispheric brain regions. A number of reported brain functional MRI (fMRI) studies have shown altered brain activity and connectivity in PTSD in other cortical regions, including the ventromedial prefrontal cortex (vmPFC) and insula^9–12^, the posterior cingulate cortex and the right frontal cortex^13^, and the rostral anterior cingulate cortex/vmPFC^14^. While our EEG lead placements (conforming to traditional AASM-accredited sleep laboratory guidelines) do not have the spatial resolution to confirm the previously identified regions, the cross-hemispheric nature of the relationships may indicate alterations in long-range cortico-cortical networks in PTSD.

Our study also found several interesting relationships between awake and sleep states with regard to coherence in PTSD. Other studies have typically examined subjects in the awake state, some using task-based approaches and others in a state of quiet wakefulness as in resting state functional connectivity studies. Using magneto-encephalography (MEG), which has a much higher temporal resolution compared with fMRI, Georgopoulos, *et al*.^15^ reported significantly altered activity and synchronous neural interactions in PTSD patients compared with control subjects. In another study with MEG in a task-free rest state, significant changes in synchronous correlations were reported between the parietal, temporal, and central regions in patients with PTSD (*n* = 80) compared to a control (*n* = 284) group^16^. Using EEG, Lee *et al*.^17^ reported that resting-state functional connectivity of *n* = 33 PTSD patients showed a decrease compared to control group (*n* = 30), and these functional connectivity measures were significantly correlated with PTSD symptom severity. More recent studies by Dunkley *et al*.^18,19^, using MEG, have reported changes in the functional coherent activity in canonical frequency bands (e.g., theta, gamma) and among various regions of the brain. Specifically, Dunkley *et al*.^18^ reported frequency-specific changes in phase-synchronized coherence within and between intrinsic networks (default-mode, salience, visual, and attention networks) during resting-state in a PTSD population and a trauma-exposed control group. They showed a clear alternation of these phase coherence in PTSD compared with control group and that such coherences were associated with PTSD symptoms. Collectively, these studies support our findings of abnormal coherent oscillations in PTSD, and that the level of such abnormality might contribute to the severity of the symptoms of the disorder.

Using sleep state as a factor in our analysis is innovative and to our knowledge, has not yet been studied. Sleep disturbances are highly prevalent in individuals with PTSD and are arguably a hallmark feature of the disorder^20–31^. It is not surprising that we found changes in EEG coherence during NREM sleep. As not all subjects exhibited REM sleep during the polysomnography, REM sleep was not analyzed, thus it remains unknown whether REM sleep, a state that has been identified as abnormal in PTSD, could contribute to our biomarker^39,40^. Our inclusion of the awake period, specifically the period just before sleep onset, may be useful for future studies utilizing a very short duration of sleep, such as a brief nap, which could potentially be enough to calculate *PTSD*_*dx*_ and *PTSD*_*sev*_ neuromarkers in the clinic. This would provide a more accessible opportunity than overnight polysomnography for the objective assessment of PTSD disease course.

Strengths of our approach include using a large cohort of individuals with gold standard overnight polysomnography, with and without PTSD that were matched for age and sleep apnea status. Other strengths include innovating on an established fact that abnormalities in brain coherence have already been reported in individuals with PTSD, yet none have identified a highly sensitive and specific marker using sleep stages as we have done. Objective neuromarkers for PTSD based in brain mechanisms are badly needed to help us understand alterations in brain circuitry and identify ways to heal or improve neurophysiological functioning.

Limitations of this study include those common to retrospective studies, namely, analysis of a sample of convenience in which PSG was clinically indicated, which could lend bias towards subjects with more sleep abnormalities compared to the general population. Our cohort was also enriched for sleep disorders, being recruited from a sleep clinic. We mitigated this issue by excluding subjects with moderate to severe sleep apnea and matching for age. Other limitations of our study included caveats that come with any correlational study, including inference of causality between these variables. It is possible that a third independent variable, such as comorbid depression, could influence the relationship between neuromarkers and symptom severity. Future analysis could further explore potential mediators and moderators between these relationships. A final limitation of this study is that the entirety of the dataset from the control and PTSD subjects was used to compute and evaluate the neuromarkers. Ideally, in order to establish the true sensitivity and specificity of the neuromarkers, training and validation datasets should be from separate cohorts. Future studies with a larger sample size would allow for more rigorous validation.

## Supplementary information

Supplementary Information

## Data Availability

Because there remains the possibility of deductive disclosure of subjects with unusual characteristics, the final, complete dataset will only be available to users under a VA-approved data-sharing agreement that provides for: (1) a commitment to using the data only for research purposes and not to identify any individual participant; (2) a commitment to securing the data using appropriate computer technology; and (3) a commitment to destroying or returning the data after analyses are completed.
