# Supplementary material for: Strong correlation of novel sleep electroencephalography coherence markers with diagnosis and severity of posttraumatic stress disorder
Source: Sci Rep. 2019 Mar 12;9:4247. doi: 10.1038/s41598-018-38102-4 (PMC6414519; doi:10.1038/s41598-018-38102-4)
Supplement: Supplementary file 1 — Supplementary Information [file 41598_2018_38102_MOESM1_ESM.docx]

**Strong correlation of novel sleep electroencephalography coherence markers with diagnosis and severity of posttraumatic stress disorder**

Mo H. Modarres^1^, Ryan A. Opel^2^, Kristianna B. Weymann^2,3^, Miranda M. Lim^2,4^

^1^Brain Rehabilitation Research Center, Malcom Randall VA Medical Center, Gainesville, Florida, USA

^2^Sleep Disorders Clinic, VA Portland Health Care System, Portland, Oregon, USA

^3^School of Nursing, Oregon Health & Science University, Portland, Oregon, USA

^4^Department of Neurology; Department of Behavioral Neuroscience; Department of Medicine; Oregon Institute of Occupational Health Sciences; Oregon Health & Science University, Portland, OR, USA

Corresponding author:

Miranda M. Lim, M.D., Ph.D.

VA Portland Health Care System

3710 SW US Veterans Hospital Road

Mail code P3-RD42

Portland, OR 97239

Phone: (503) 220-8262 x57404

Fax: (503) 273-5351

[lmir@ohsu.edu](mailto:lmir@ohsu.edu)

**Supplementary Material**

## Supplementary Table S1. Diagnostic *BCMr* Markers (PTSD_dx_) in Wakefulness

## Statistical comparison of the eight *BCMr* markers in Control and PTSD groups.

## Supplementary Table S2. Diagnostic *BCMr* Markers (PTSD_dx_) in N1 and N2 Sleep

## Statistical comparison of the two *BCMr* markers in Control and PTSD groups.

## Supplementary Table S3. *BCMr* marker Components of PTSD Symptom Severity Marker (PTSD_sev_) in Wakefulness

Statistics of *BCMr_1_* to *BCMr_5_* markers that were linearly combined to produce *PTSD_sev_*_­_.

## Supplementary Table S4. *BCMr* marker Components of PTSD Symptom Severity Marker (PTSD_sev_) in N1 and N2 Sleep

Statistics of *BCMr_1_* and *BCMr_2_* markers that were linearly combined to produce *PTSD_sev_*.

## Supplementary Table S1

| **Sleep Stage** | **Formula** | **Control (n=38)** | **PTSD (n=38)** | ***P Value*** | **Diagram for *PTSD_dx_*** |
| --- | --- | --- | --- | --- | --- |
| W | ${BCMr}_{1}=\frac{\boldsymbol{BCM} \left( O1,O2, \left[ 0.6-1.6 \right], W \right)}{\boldsymbol{BCM}\left( F3,F4, \left[ 0.2-1.2 \right], W \right)}$ | 0.73 ± 0.33 | 1.14 ± 0.47 | < 0.0001 | **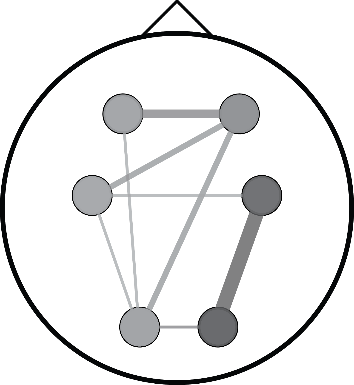** |
| W | ${BCMr}_{2}=\frac{\boldsymbol{BCM} \left( C3, C4, \left[ 22.8 -23.8 \right], W \right)}{\boldsymbol{BCM}\left( F3,F4, \left[ 16.8-17.8 \right], W \right)}$ | 1.08 ± 0.38 | 0.82 ± 0.27 | <0.001 |  |
| W | ${BCMr}_{3}=\frac{\boldsymbol{BCM} \left( F3, F4, \left[ 19.0 -20.0 \right], W \right)}{\boldsymbol{BCM}\left( O2,C4, \left[ 18.6-19.6 \right], W \right)}$ | 0.46 ± 0.44 | 0.77 ± 0.50 | < 0.004 |  |
| W | ${BCMr}_{4}=\frac{\boldsymbol{BCM} \left( O1, C3, \left[ 6.6 -7.6 \right], W \right)}{\boldsymbol{BCM}\left( C3,F4, \left[ 45.8-46.8 \right], W \right)}$ | 4.34 ± 1.80 | 2.71 ± 1.71 | <0.0001 |  |
| W | ${BCMr}_{5}=\frac{\boldsymbol{BCM} \left( O2, C4, \left[ 43.4 -44.4 \right], W \right)}{\boldsymbol{BCM}\left( O2,C4, \left[ 27.0-28.0 \right], W \right)}$ | 1.05 ± 0.16 | 1.23 ± 0.21 | < 0.0001 |  |
| W | ${BCMr}_{6}=\frac{\boldsymbol{BCM} \left( O2, C4, \left[ 9.6- 10.6 \right], W \right)}{\boldsymbol{BCM}\left( O1, F3, \left[ 7.2-8.2 \right], W \right)}$ | 1.28 ± 0.48 | 1.72 ± 0.47 | < 0.001 |  |
| W | ${BCMr}_{7}=\frac{\boldsymbol{BCM} \left( O2, C4, \left[ 6.4- 7.4 \right], W \right)}{\boldsymbol{BCM}\left( O1, F4, \left[ 45.6-46.6 \right], W \right)}$ | 4.16 ± 1.76 | 2.51 ± 1.47 | <0.0001 |  |
| W | ${BCMr}_{8}=\frac{\boldsymbol{BCM} \left( O1, C4, \left[ 9.2- 10.2 \right], W \right)}{\boldsymbol{BCM}\left( C3, F4, \left[ 17.6-18.6 \right], W \right)}$ | 1.71 ± 0.80 | 1.07 ± 0.48 | <0.0001 |  |

**Supplementary Table S2**

| **Sleep Stage** | **Formula** | **Control (n=38)** | **PTSD (n=38)** | ***P* Value** | **Diagram for *PTSD_dx_*** |
| --- | --- | --- | --- | --- | --- |
| N1 | ${BCMr}_{1}=\frac{\boldsymbol{BCM} \left( F3, F4, \left[ 7.8-8.8 \right], N1 \right)}{\boldsymbol{BCM}\left( C3,F4, \left[ 7.4-8.4 \right], N1 \right)}$ | 1.02 ± 0.31 | 1.40 ± 0.36 | <0.0001 | 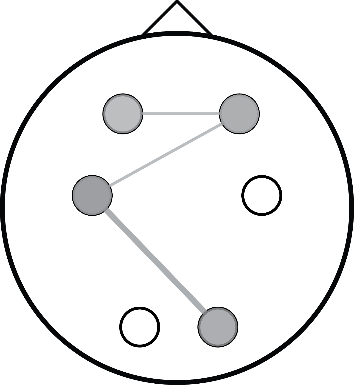 |
| N1 | ${BCMr}_{2}=\frac{\boldsymbol{BCM} \left( O2, C3, \left[ 32.0 -33.0 \right], N1 \right)}{\boldsymbol{BCM}\left( O2, C3, \left[ 32.8 -33.8 \right], N1 \right)}$ | 1.04 ± 0.06 | 0.98 ± 0.06 | <0.0001 |  |
| N2 | ${BCMr}_{1}=\frac{\boldsymbol{BCM} \left( C3, C4, \left[ 7.0-8.0 \right], N2 \right)}{\boldsymbol{BCM}\left( F3,F4, \left[ 8.6-9.6 \right], N2 \right)}$ | 1.17 ± 0.62 | 0.77 ± 0.19 | <0.0004 | **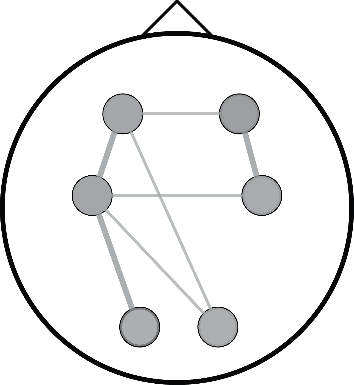** |
| N2 | ${BCMr}_{2}=\frac{\boldsymbol{BCM} \left( O1, C3, \left[ 32.4 -33.4 \right], N2 \right)}{\boldsymbol{BCM}\left( O1, C3, \left[ 32.0-33.0 \right], N2 \right)}$ | 0.99 ± 0.01 | 1.01 ± 0.02 | <0.0002 |  |
| N2 | ${BCMr}_{3}=\frac{\boldsymbol{BCM} \left( C3, F3, \left[ 36.2 -37.2 \right], N2 \right)}{\boldsymbol{BCM}\left( C3, F3, \left[ 35.6-36.6 \right], N2 \right)}$ | 1.0 ± 0.01 | 0.97 ± 0.01 | <0.009 |  |
| N2 | ${BCMr}_{4}=\frac{\boldsymbol{BCM} \left( C4, F4, \left[ 16.2 -17.2 \right], N2 \right)}{\boldsymbol{BCM}\left( C4, F4, \left[ 12.8-13.8 \right], N2 \right)}$ | 1.01 ± 0.07 | 0.96 ± 0.07 | <0.0007 |  |
| N2 | ${BCMr}_{5}=\frac{\boldsymbol{BCM} \left( O2, C3, \left[ 10.8-11.8 \right], N2 \right)}{\boldsymbol{BCM}\left( O2,F3, \left[ 10.2-11.2 \right], N2 \right)}$ | 0.94 ± 0.16 | 1.22 ± 0.39 | <0.0002 |  |

## Supplementary Table S3

| **Sleep Stage** | **Formula** | **PTSD (n=38)** | ***P* Value** | **Diagram for *PTSD_sev_*** |
| --- | --- | --- | --- | --- |
| W | ${BCMr}_{1}=\frac{\boldsymbol{BCM} \left( C3, F4, \left[ 0.8-1.8 \right], W \right)}{\boldsymbol{BCM}\left( C4,F4, \left[ 7.4-8.4 \right], W \right)}$ | 0.91 ± 0.15 | < 0.02 | 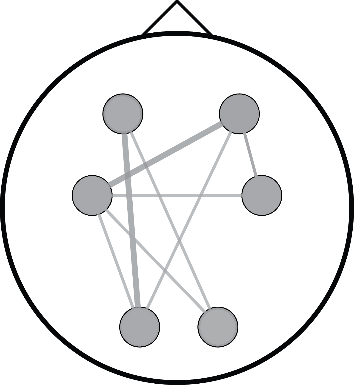 |
| W | ${BCMr}_{2}=\frac{\boldsymbol{BCM} \left( O1, C3, \left[ 0.6 -1.6 \right], W \right)}{\boldsymbol{BCM}\left( O1, F3, \left[ 6.8-7.8 \right], W \right)}$ | 1.69 ± 0.60 | <0.05 |  |
| W | ${BCMr}_{3}=\frac{\boldsymbol{BCM} \left( O1, C4, \left[ 20.0 -21.0 \right], W \right)}{\boldsymbol{BCM}\left( O1, F3, \left[ 45.6-46.6 \right], W \right)}$ | 0.56 ± 0.28 | <0.005 |  |
| W | ${BCMr}_{4}=\frac{\boldsymbol{BCM} \left( C3, F4, \left[ 20.4 -21.4 \right], W \right)}{\boldsymbol{BCM}\left( O2, F3, \left[ 40.0-41.0 \right], W \right)}$ | 1.04 ± 0.34 | <0.00001 |  |
| W | ${BCMr}_{5}=\frac{\boldsymbol{BCM} \left( C3, C4, \left[ 20.0 -21.0 \right], W \right)}{\boldsymbol{BCM}\left( O2,C3, \left[ 6.8-7.8 \right], W \right)}$ | 1.25 ± 0.67 | < 0.00001 |  |

## Supplementary Table S4

| **Sleep Stage** | **Formula** | **PTSD (n=38)** | ***P* Value** | **Diagram for *PTSD_sev_*** |
| --- | --- | --- | --- | --- |
| N1 | ${BCMr}_{1}=\frac{\boldsymbol{BCM} \left( C3, F4, \left[ 40.8-41.8 \right], N1 \right)}{\boldsymbol{BCM}\left( C4,F3, \left[ 41.8-42.8 \right], N1 \right)}$ | 1.03 ± 0.14 | < 0.0001 | 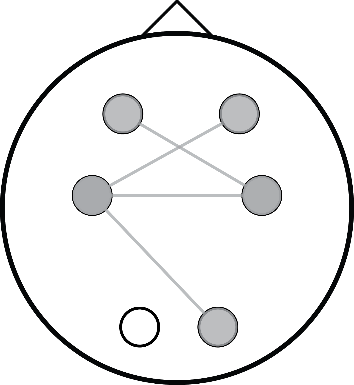 |
| N1 | ${BCMr}_{2}=\frac{\boldsymbol{BCM} \left( C3, C4, \left[ 20.6 -21.6 \right], N1 \right)}{\boldsymbol{BCM}\left( O2, C3, \left[ 8.0-9.0 \right], N1 \right)}$ | 1.42 ± 0.92 | <0.00001 |  |
| N2 | ${BCMr}_{1}=\frac{\boldsymbol{BCM} \left( O1, C4, \left[ 20.0-21.0 \right], N2 \right)}{\boldsymbol{BCM}\left( O2,F4, \left[ 48.2-49.2 \right], N2 \right)}$ | 0.74 ± 0.44 | < 0.0005 | 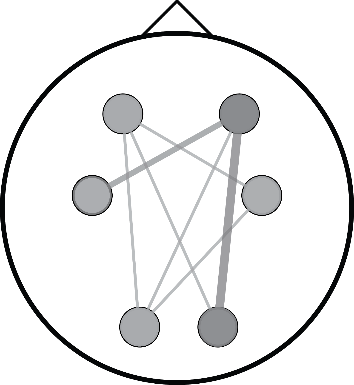 |
| N2 | ${BCMr}_{2}=\frac{\boldsymbol{BCM} \left( O1, F3, \left[ 19.2-20.2 \right], N2 \right)}{\boldsymbol{BCM}\left( O2, F4, \left[ 24.0-25.0 \right], N2 \right)}$ | 0.91 ± 0.23 | <0.005 |  |
| N2 | ${BCMr}_{3}=\frac{\boldsymbol{BCM} \left( O1, F4, \left[ 20.8 -21.8 \right], N2 \right)}{\boldsymbol{BCM}\left( O2, F4, \left[ 22.6-23.6 \right], N2 \right)}$ | 0.80 ± 0.29 | <0.0005 |  |
| N2 | ${BCMr}_{4}=\frac{\boldsymbol{BCM} \left( C3, F4, \left[ 37.8 -38.8 \right], N2 \right)}{\boldsymbol{BCM}\left( C4 F3, \left[ 39.6-40.6 \right], N2 \right)}$ | 1.04 ± 0.34 | <0.00001 |  |
| N2 | ${BCMr}_{5}=\frac{\boldsymbol{BCM} \left( C3, F4, \left[ 19.6 -20.6 \right], N2 \right)}{\boldsymbol{BCM}\left( O2,F3, \left[ 10.0-11.0 \right], N2 \right)}$ | 1.89 ± 1.37 | < 0.00001 |  |
